# Regulation of Human Platelet Activation and Prevention of Arterial Thrombosis in Mice by Auraptene through Inhibition of NF-κB Pathway

**DOI:** 10.3390/ijms21134810

**Published:** 2020-07-07

**Authors:** Chih-Wei Hsia, Ming-Ping Wu, Ming-Yi Shen, Chih-Hsuan Hsia, Chi-Li Chung, Joen-Rong Sheu

**Affiliations:** 1 Graduate Institute of Medical Sciences, College of Medicine, Taipei Medical University, Taipei 110, Taiwan; d119106003@tmu.edu.tw (C.-W.H.); mpwu@mail.chimei.org.tw (M.-P.W.); T014913@ms.skh.org.tw (C.-H.H.); 2 Department of Obstetrics and Gynecology, Chi-Mei Medical Center, Tainan 710, Taiwan; 3 Graduate Institute of Biomedical Sciences, China Medical University, Taichung 404, Taiwan; shenmy1124@gmail.com; 4 Translational Medicine Center, Shin Kong Wu Ho-Su Memorial Hospital, Taipei 111, Taiwan; 5 Division of Pulmonary Medicine, Department of Internal Medicine, Taipei Medical University Hospital, Taipei 110, Taiwan; 6 Department of Pharmacology, College of Medicine, Taipei Medical University, Taipei 110, Taiwan

**Keywords:** arterial thrombosis, auraptene, human platelet, hydroxyl radical, NF-κB, PLCγ2-PKC activation

## Abstract

Platelets are major players in the occurrence of cardiovascular diseases. Auraptene is the most abundant coumarin derivative from plants, and it has been demonstrated to possess a potent capacity to inhibit platelet activation. Although platelets are anucleated cells, they also express the transcription factor, nuclear factor-κB (NF-κB), that may exert non-genomic functions in platelet activation. In the current study, we further investigated the inhibitory roles of auraptene in NF-κB-mediated signal events in platelets. MG-132 (an inhibitor of proteasome) and BAY11-7082 (an inhibitor of IκB kinase; IKK), obviously inhibited platelet aggregation; however, BAY11-7082 exhibited more potent activity than MG-132 in this reaction. The existence of NF-κB (p65) in platelets was observed by confocal microscopy, and auraptene attenuated NF-κB activation such as IκBα and p65 phosphorylation and reversed IκBα degradation in collagen-activated platelets. To investigate cellular signaling events between PLCγ2-PKC and NF-κB, we found that BAY11-7082 abolished PLCγ2-PKC activation; nevertheless, neither U73122 nor Ro31-8220 had effect on NF-κB activation. Furthermore, both auraptene and BAY11-7082 significantly diminished HO• formation in activated platelets. For in vivo study, auraptene prolonged the occlusion time of platelet plug in mice. In conclusion, we propose a novel inhibitory pathway of NF-κB-mediated PLCγ2-PKC activation by auraptene in human platelets, and further supported that auraptene possesses potent activity for thromboembolic diseases.

## 1. Introduction

Cardiovascular diseases (CVDs) are the largest cause of death globally. CVDs are often caused by thrombotic events such as coronary heart disease. Platelets are major players in the occurrence of CVDs since they are involved in various thrombo-inflammatory diseases, particularly atherosclerosis and its progression to atherothrombosis in acute coronary syndrome (ACS) patients [1]. In fact, platelets control key hemostasis and formation of thrombosis, nevertheless, thrombosis can become pathological when it occurs, predominantly after the rupture of an atherosclerotic plaque. Thus, following an atherosclerotic vascular lesion, exposed vascular subendothelial connective tissues (such as collagen) in conjunction with thrombin and ADP production, triggers platelet activation. Subsequently, the inside-out signaling of the integrin α_IIb_β_3_, in response to platelet activation, leads to the binding of fibrinogen, promoting the formation of a thrombus [2]. Such a process could induce a partial or complete occlusion of the blood vessel, which leads to a decrease or blockage of the blood flow and thus becomes a cause of occurrence of ischemia or infarction of an irrigated organ such as the heart [3].

The NF-κB signaling system is a central coordinator of a diverse array of cellular actions including immune/inflammatory reactions, carcinogenesis, neurodegenerative processes and vascular responses [4]. NF-κB, as “NF” (nuclear factor) implies, works in the capacity of a transcription factor, which upon activation is translocated into the nucleus to transcriptionally regulate nuclear gene expression. Under basal conditions, NF-κB is bound by inhibitor of κB (IκB) to be retained in the cytosol where it remains inactive. Of the two subunits (IκBα and IκBβ), IκBα is the most represented. Upon activation, the IκB kinase (IKK) complex phosphorylates IκB causing its proteasome-mediated degradation, releasing NF-κB for nuclear translocation to exert its roles as a transcription factor for transcription of pro-inflammatory and pro-survival genes [1]. In such cellular settings, the role of NF-κB is genomic and well documented in nucleated cells. Among IKK subunits (α, β, and γ), IKKβ is the most active. Thus, most, if not all, actions of NF-κB have been ascribed to nuclear events. In addition, three IKK family members are also expressed in platelets, β form being even more strongly expressed in platelets than either α or γ form [5,6]. NF-κB, including p65 and p50 subunits, is also present in anucleated platelets and its function has also been studied [7,8,9,10], whereas it is still not completely clear especially its relationship with other signal pathways in platelet activation. The question remains us to check whether or not this transcription factor is functionally present in a novel way, unrelated to transcriptional regulation in platelets.

Auraptene is the best-known and most abundant prenyloxycoumarin present in nature. It was first isolated from members of the genus *Citrus*. Auraptene has shown a remarkable effect in the prevention of degenerative diseases. In view of natural coumarin derivatives such as warfarin clinical use as an anticoagulant agent [11]; however, it is important to highlight that anticoagulants belonging to the dicoumarols group (i.e., warfarin) owe its effect to the inhibition of the enzyme epoxide reductase vitamin K. It catalyzes the step of reduced active. Such mechanism does not correlate with the monocoumarins (i.e., esculetin), which have no direct or indirect effect on coagulation [12]. Many studies have reported the effect of auraptene as a chemopreventative agent against cancers of liver, skin, tongue, esophagus, and colon in rodent models [13]. In our previous report [14], we found that auraptene markedly inhibited collagen (1 μg/mL)-stimulated human platelet activation, and it IC_50_ was approximated at 35 μM. However, the inhibition was not significantly stimulated with either arachidonic acid, thrombin, or U46619. We suggested the mechanisms of auraptene in platelet activation may be through interference with the phosphorylation of PLCγ2-PKC cascade stimulated by collagen. Platelets are anucleated, do not differentiate or proliferate, and thus are a good model for studying nongenomic functions of NF-κB in cells. Therefore, our previous findings led us to conduct a thorough investigation of the role/relationship of the auraptene-mediated signaling pathway between NF-κB with PLCγ2-PKC in platelet activation.

## 2. Results

### 2.1. Inhibitory Profiles of Auraptene and NF-κB Inhibitors in Human Platelet Aggregation and ATP-Release Reaction Stimulated by Collagen

**Figure 1:**
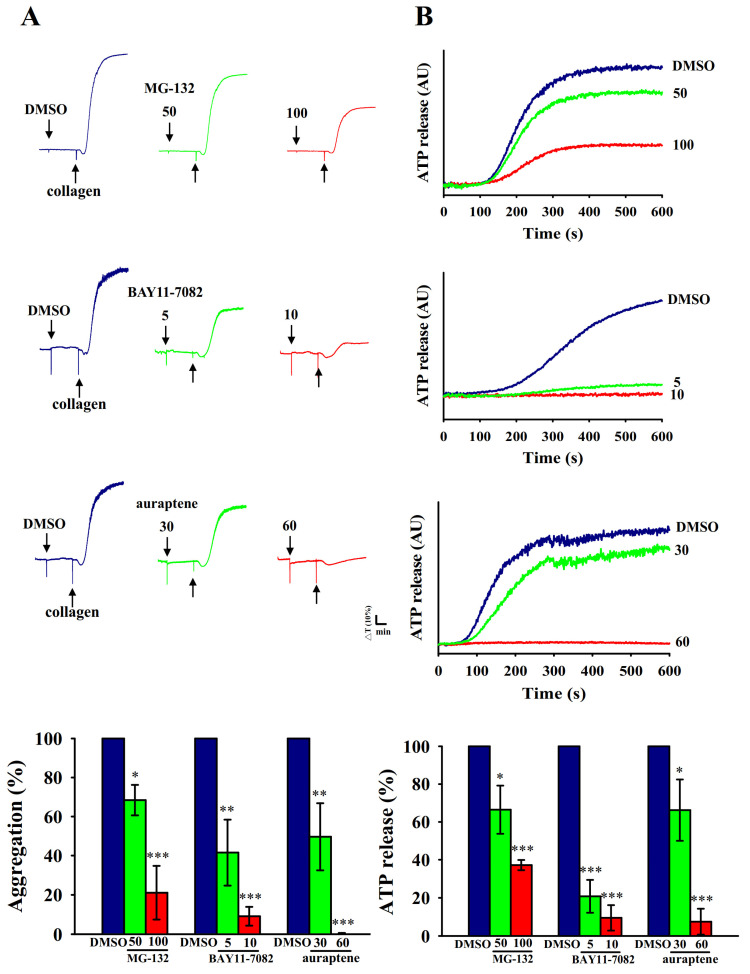
Inhibitory profiles of NF-κB inhibitors and auraptene in platelet aggregation and ATP-release reaction stimulated by collagen in human platelets. Washed human platelets (3.6 × 10^8^ cells/mL) were preincubated with MG-132 (50 and 100 μM), BAY11-7082 (5 and 10 μM) or auraptene (30 and 60 μM), followed by the addition of collagen (1 μg/mL) to trigger (**A**) platelet aggregation and (**B**) ATP-release reaction (AU; arbitrary unit). The corresponding statistical data are displayed on the below panel of each figure. Data are presented as mean ± SEM (*n* = 4). * *p* < 0.05, ** *p* < 0.01, and *** *p* < 0.001, compared with the 0.1% DMSO-treated group.

MG-132 is a proteasome inhibitor through blocking activation of NF-κB by preventing proteasome-mediated degradation of IκB, an endogenous inhibitor of NF-κB [15]. Furthermore, BAY11-7082 is a representative IKK inhibitor that has pharmacological activities that include anticancer, neuroprotective, and anti-inflammatory effects [16]. In this study, both MG-132 (50 and 100 μM) and BAY11-7082 (5 and 10 μM) or auraptene (30 and 60 μM) concentration-dependently inhibited platelet aggregation stimulated by collagen (1 μg/mL) in human platelets (Figure 1A). BAY11-7082 exhibited more potent activity than MG-132 at inhibiting platelet aggregation on a molar basis. Moreover, platelet activation is associated with the release of granular contents (e.g., ATP from dense granules), thus causing abundant platelet aggregation. As shown in Figure 1B, BAY11-7082 also exhibited more potent activity than MG-132 at inhibiting ATP-release reaction under the same condition. Therefore, BAY11-7082 was used to further explore the relationship between NF-κB with PLCγ2-PKC cascade in auraptene-mediated antiplatelet activity for the following experiments.

### 2.2. Platelet Activation Triggers NF-κB Signals 

**Figure 2:**
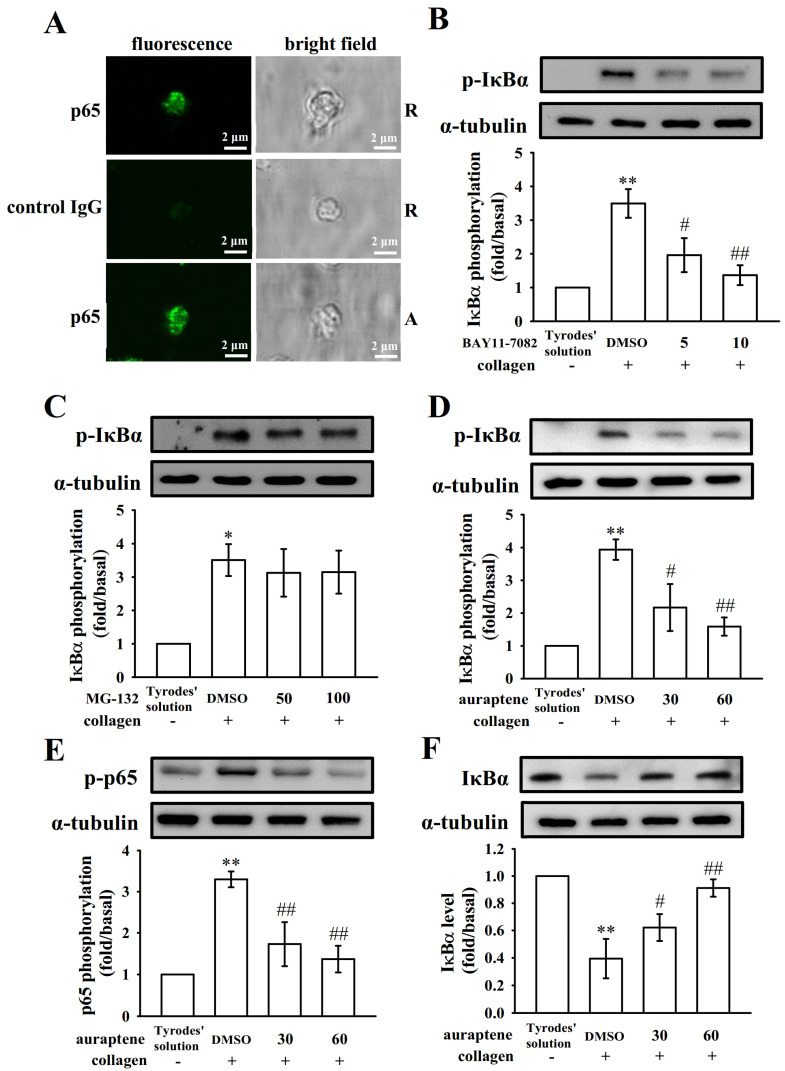
Effects of NF-κB activation by BAY11-7082, MG-132, and auraptene in platelets. (**A**) The confocal image (10 × 100 magnification) of NF-κB (p65) in resting (R) or collagen-activated (A) platelets. p65 or control IgG was labeled with goat anti-rabbit IgG-conjugated FITC (shown in green color) as described in Materials and Methods. For other experiments, washed platelets were preincubated with a solvent control (0.1% DMSO), BAY11-7082 (5 and 10 μM), MG-132 (50 and 100 μM), or auraptene (30 and 60 μM), followed by the addition of collagen (1 μg/mL) to trigger (**B**–**D**) IκBα and (**E**) p65 phosphorylation, or (**F**) IκBα protein degradation. Profiles (**A**) are representative examples of four similar experiments. The corresponding statistical data displayed in B–F. Data are presented as mean ± SEM (*n* = 4). * *p* < 0.05 and ** *p* < 0.01 compared with the resting control (Tyrode’s solution); ^#^ *p* < 0.05 and ^##^ *p* < 0.01, compared with the 0.1% DMSO-treated group.

Pleiotropic NF-κB normally exists as an inactive cytoplasmic complex, the predominant form of which is a heterodimer composed of p50 and p65 subunits tightly bound to inhibitory proteins of the IκB family [17]. As shown in the Figure 2A, NF-κB was confirmed by immunofluorescent stained with the anti-p65 mAb (green fluorescence) compared with isotype control IgG in resting or collagen-activated using confocal laser fluorescence microscopy. We found that there are no significant differences in fluorescence intensity between two groups (Figure 2A). Figure 2B-F demonstrated the occurrence of NF-κB activation in anucleated platelets, either IκBα or p65 phosphorylation, or IκBα protein degradation were significantly increased after being stimulated by collagen (1 μg/mL). Furthermore, BAY11-7082 (5 and 10 μM) exhibited more potent activity than MG-132 (50 and 100 μM) in inhibiting collagen-induced IκBα phosphorylation (Figure 2B–C). Pretreatment with auraptene (30 and 60 μM) obviously attenuated IκBα and p65 phosphorylation and reversed IκBα protein degradation after collagen stimulation (Figure 2D–F). Compiled data are shown in Figure 2B–F under the images. These results suggest the inhibition of NF-κB signals may play a crucial mechanism in regulation of platelet activation by auraptene.

### 2.3. The Relationship between NF-κB Signaling and PLCγ2-PKC Activation in Human Platelets

**Figure 3:**
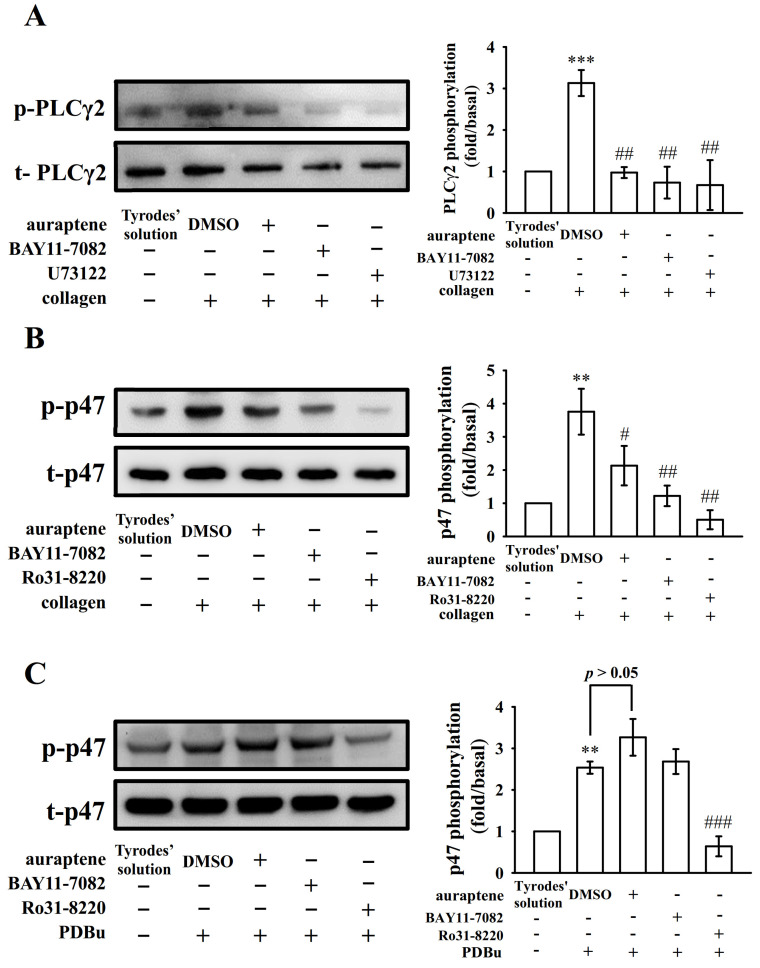
Regulatory effects of NF-κB inhibitors and auraptene on PLCγ2 phosphorylation and PKC activation. Washed platelets were preincubated with 0.1% DMSO, auraptene (60 μM), BAY11-7082 (10 μM), U73122 (5 μM), or Ro31-8220 (2 μM) and then treated with collagen (1 μg/mL) or PDBu (150 nM) to trigger either (**A**) PLCγ2 phosphorylation or (**B**,**C**) PKC activation (p-p47). The corresponding statistical data are displayed on the right panel of each figure. Data are given as means ± SEM (*n* = 4). ** *p* < 0.01 and *** *p* < 0.001, compared with the resting platelets (Tyrode’s solution); ^#^ *p* < 0.05, ^##^ *p* < 0.01, and ^###^ *p* < 0.001, compared with the 0.1% DMSO-treated group.

**Figure 4:**
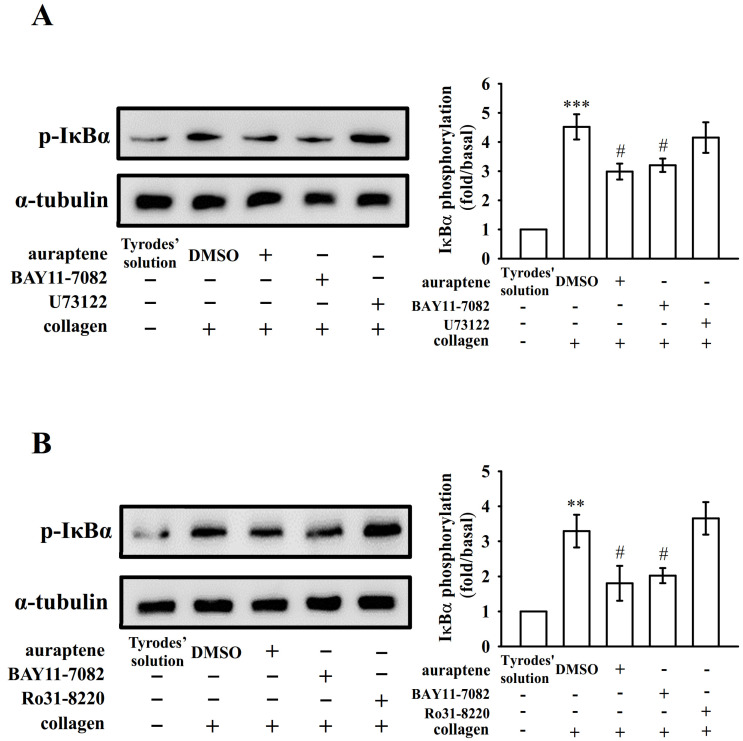
Regulatory profiles of auraptene and PLC/PKC inhibitors on IκBα protein degradation. Washed platelets were preincubated with 0.1% DMSO, auraptene (60 μM), BAY11-7082 (10 μM), U73122 (5 μM), or Ro31-8220 (2 μM) and then treated with collagen (1 μg/mL) to trigger (**A**,**B**) IκBα protein phosphorylation. The corresponding statistical data are displayed on the right panel of each figure. Data are given as means ± SEM (*n* = 4). ** *p* <0.01 and *** *p* < 0.001, compared with the resting platelets (Tyrode’s solution); ^#^ *p* < 0.05, compared with the 0.1% DMSO-treated group.

PLC comprise a family of kinases that hydrolyze phosphatidylinositol 4,5-bisphosphate [PI(4,5)P2] to produce two second messengers, diacylglycerol (DAG) and inositol trisphosphate (IP_3_). DAG activates PKC-stimulating protein phosphorylation (p47 protein; pleckstrin) and ATP release in activated platelets; IP_3_ elevates calcium influx [18]. In previous study [14], auraptene markedly diminished the PLCγ2 phosphorylation and PKC activation in collagen-activated platelets. However, auraptene had no significant effect in PDBu (PKC activator)-induced platelet aggregation [14], indicating that auraptene inhibits platelet activation through PLCγ2/PKC cascade. To further investigate cellular signaling events between PLCγ2-PKC and NF-κB, various inhibitors were employed in the following experiments. We found that both U73122 (5 μM; an inhibitor of PLCγ2) and BAY11-7082 (10 μM) or auraptene (60 μM) nearly abolished collagen-induced PLCγ2 phosphorylation (Figure 3A). Moreover, both Ro31-8220 (2 μM; an inhibitor of PKC activation) and BAY11-7082 (10 μM) or auraptene (60 μM) obviously diminished PKC activation (p47 protein; pleckstrin) stimulated by collagen (Figure 3B), neither BAY11-7082 nor auraptene significantly affected this reaction stimulated by PDBu (150 nM) (Figure 3C). Moreover, pretreatment with auraptene (60 μM) and BAY11-7082 (10 μM) (Figure 4A), but not U73122 (5 μM) or Ro31-8220 (2 μM) (Figure 4B), significantly diminished IκBα phosphorylation stimulated by collagen, indicating that NF-κB seems to be an upstream regulator of PLCγ2-PKC activation, which involved in the auraptene-mediated antiplatelet activation.

### 2.4. Effectiveness of BAY11-7082 and Auraptene in Collagen-Stimulated Hydroxyl Radicals (HO•) Formation

**Figure 5:**
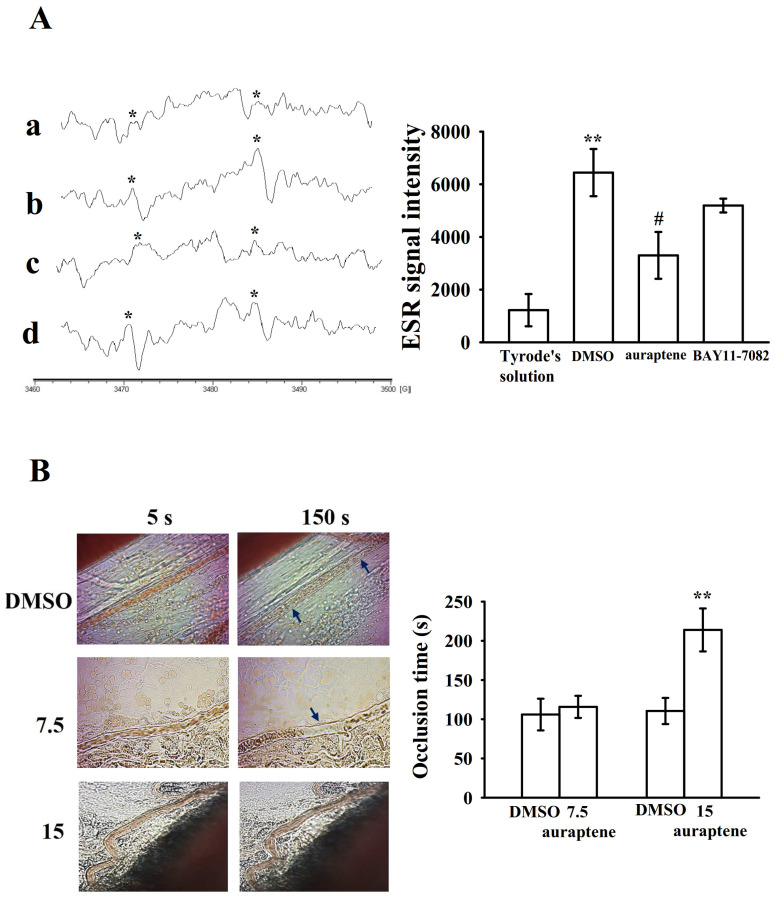
Effects of auraptene on HO• formation in human platelets and vascular thrombosis in the mesenteric venules of mice. (**A**) For the electron spin resonance (ESR) study, washed platelets were incubated with Tyrode’s solution (**a**, resting group), solvent control (**b**, 0.1% DMSO), auraptene (**c**, 30 μM), or (**d**) BAY11-7082 (10 μM), followed by the addition of collagen (1 μg/mL) to trigger HO• (hydroxyl radical) formation. An asterisk (*) indicates the formation of HO•. Spectra are representative examples of four similar experiments. Data are given as means ± SEM (*n* = 4). ** *p* < 0.01, compared with the resting platelets (Tyrode’s solution); # p < 0.05, compared with the 0.1% DMSO-treated group. (**B**) For animal study, mice were administered an intravenous bolus of the solvent control (0.1% DMSO) or auraptene (7.5 and 15 mg/kg), and the mesenteric venules were irradiated to induce microthrombus formation (occlusion time). Microscopic images (400× magnification) of 0.1% DMSO-treated groups and the auraptene (7.5 and 15 mg/kg)-treated groups were recorded at 5 and 150 s after irradiation, respectively. Data are given as means ± SEM (*n* = 8). ** *p* < 0.01, compared with the 0.1% DMSO-treated group. The photographs are representative of eight similar experiments, and the arrows indicate platelet plug formation.

Reactive oxygen species (ROS; hydrogen peroxide, hydroxyl radicals, etc.) derived from platelet activation might amplify platelet reactivity during thrombus formation. However, the regulatory pathways of ROS especially for hydroxyl radicals in platelet activation remain obscure. As shown in Figure 5A, a typical ESR signal of hydroxyl radical (HO•) formation was triggered by collagen (1 μg/mL) compared to resting platelets (Figure 5Aa,b); auraptene (30 μM) markedly reduced collagen-induced hydroxyl radical formation (Figure 5Ac); nevertheless, BAY11-7082 (10 μM) had no significant effect in this reaction (Figure 5Ad). 

### 2.5. Regulatory In Vivo Activity of Auraptene in Vascular Thrombus Formation

The antithrombotic activity of auraptene (7.5 and 15 mg/kg) was observed in experimental mice. The occlusion time in the mesenteric microvessels of mice pretreated with 15 µg/kg fluorescein sodium was approximately 120 s. The resulting occlusion times were significantly extended after 15 mg/kg auraptene treatments compared with those after 0.1% DMSO treatment (control vs. 7.5 mg/kg, auraptene 106.0 ± 20.2 s vs. 115.8 ± 14.1 s, *n* = 8, *p* > 0.05; control vs. 15 mg/kg auraptene, 110.5 ± 16.7 s vs. 213.9 ± 27.4 s, *n* = 8, *p* < 0.01; Figure 5B). After irradiation, a thrombotic platelet plug was observed in the mesenteric microvessels at 150 s, in either 0.1% DMSO- or 7.5 mg/kg auraptene-treated group (Figure 5B; left panel, arrows). On administration of 15 mg/kg auraptene, platelet plug formation was not observed at 150 s after irradiation (Figure 5B; left panel).

## 3. Discussion

Auraptene is a hydrophobic molecule—when administered orally, it undergoes rapid absorption and is rapidly distributed to all tissues. Besides, auraptene is easily available in large amounts through chemical synthesis. Therefore, auraptene is a potentially new therapeutic candidate against arterial thrombosis-related diseases for humans [14]. In the current study, auraptene potently inhibited NF-κB activation (including IκBα phosphorylation and degradation as well as p65 phosphorylation) stimulated by collagen in human platelets, indicating the NF-κB signal plays a crucial role for auraptene-mediated antiplatelet activity. In the present study, the blood was collected from individual healthy human volunteers, and hence a small difference may occur in the experimental results, for example, auraptene does not show with the same extent of inhibition of IκBα phosphorylation as shown in the Figure 2 and Figure 4. The function of NF-κB was extensively studied in nucleated cells. Diverse stimuli, including cytokines, viral infection, UV radiation, and free radicals, can induce NF-κB activation. Genes regulated by NF-κB include those involved in inflammation, cell survival, differentiation, and proliferation responses [17]. Therefore, NF-κB is an attractive target for therapeutic interventions against cancer and inflammatory diseases.

Several studies have found that anucleated cells, platelets, express several transcription factors such as peroxisome proliferator-activated receptors (PPARs) [19] and glucocorticoid receptors [20], suggesting these transcription factors can exert an activity other than genomic functions on platelets. In the current study, we also provide the evidence by confocal laser fluorescence microscopy to demonstrate the exiting of NF-κB (p65) in human platelets. The question remains as to ask whether the NF-κB is practically present in a novel way cooperated with other signal machinery in platelet activation. It has been reported that NF-κB inhibitors prevented platelet activation, such as thromboxane A2 formation, fibrinogen adhesion, and P-selectin expression [8]. In the present study, we further found that pretreatment with BAY11-7082, an irreversible inhibitor of IκBα phosphorylation, obviously exhibited more potent activity than MG-132 at inhibiting both platelet aggregation and ATP-release reaction, providing clearly evidence that NF-κB participates in various steps of platelet activation.

PLCγ2 phosphorylation is involved in collagen-dependent signaling in platelets [21]. Previous study has implied that auraptene-mediated inhibition of platelet aggregation occurs through a PLCγ2-PKC dependent mechanism [14]. In the present study, BAY11-7082 obviously attenuated both PLCγ2 phosphorylation and PKC activation stimulated by collagen, but had no effects on PKC activation stimulated by PDBu. In contrast, U73122 did not significantly reduce IκBα phosphorylation stimulated by collagen. Taken together, these results indicate that NF-κB is upstream regulator of PLCγ2-PKC activation in human platelets.

Some of the hydrogen peroxide produced by platelets is converted into hydroxyl radicals, which involve in the initial phase of platelet activation [22]. The results from ESR study pointed out that auraptene but not BAY11-7082 scavenges HO• formation in activated platelets. Taken together, we hypothesized that the NF-κB signal acts as an upstream regulator for the PLCγ2-PKC activation, nevertheless, it seems unable to regulate the hydroxyl radical formation in activated platelets. However, our study does not rule out the possibility that other, yet-unidentified signals are involved in NF-κB-mediated inhibition of platelet activation by auraptene.

Exposure of subendothelial collagen triggers platelet adhesion and aggregation at the site of vascular endothelial cell injury, followed by arterial thrombus formation. A previous study [14] demonstrated that auraptene at 7.5 and 15 mg/kg significantly reduced the mortality of ADP-induced acute pulmonary thromboembolism in mice. In the current study, we further confirmed that auraptene markedly prolonged the occlusion time in mesenteric microvessels, which were continuously irradiated with fluorescein sodium throughout the experimental period, leading to strong damage to endothelial cells. Thus, a part of the reason for auraptene-mediated inhibition of thrombogenesis in vivo may be its free radical scavenging activity.

Herein, our study on platelet NF-κB might help to characterize the exceptional function of this protein in platelets. Although emerging studies corroborate that NF-κB has a primordial role to a positive regulation of platelet activation, further investigations are warranted to fully elucidate the mechanics of NF-κB, especially that another perplexing and equally interesting extra-platelet function for platelet NF-κB has recently been fading in and since few studies showcase an opposite role for NF-κB in platelet function and may, therefore, act as a double-edged sword [10,23]. This, and given the fact that the targeting of NF-κB by auraptene is elucidated to ameliorate diverse pathophysiological conditions, more pre-clinical research might also bestow upon platelet NF-κB a therapeutic potential in cardiovascular diseases. Thus, inhibiting platelet NF-κB-mediated PLCγ2-PKC activation may have a high therapeutic potential to treat thrombotic disorders.

## 4. Materials and Methods

### 4.1. Materials

Auraptene (>98%), MG-132, and U73122 were purchased from Cayman Chem. (Ann Arbor, MI, USA). Luciferin/luciferase, collagen (type I), dimethyl sulfoxide (DMSO), bovine serum albumin (BSA), prostaglandin E_1_ (PGE_1_), heparin, 5,5-dimethyl-1 pyrroline N-oxide (DMPO), (E)-3-(4-methylphenylsulfonyl)-2-propenenitrile (BAY11-7082), Ro31-8220, and phorbol-12, 13-dibutyrate (PDBu) were purchased from Sigma (St Louis, MO, USA). Anti-IκBα (44D4) monoclonal antibody (mAb), anti-phospho-IκBα (Ser^32/36^) (5A5), anti-NF-κB p65 mAbs, and anti-phospho-NF-κB p65 (Ser^536^) polyclonal antibody (pAb), anti-phospholipase C (PLC)γ2, anti-phospho (Tyr^759^) PLCγ2 pAbs were all from Cell Signaling (Beverly, MA, USA). An anti-pleckstrin (p47) pAb was purchased from Genetex (Irvine, CA, USA). The anti-α-tubulin mAb was from NeoMarkers (Fremont, CA, USA); and the Hybond-P polyvinylidene difluoride (PVDF) membrane, enhanced chemiluminescence (ECL) Western blotting detection reagent and analysis system, horseradish peroxidase (HRP)-conjugated donkey anti-rabbit immunoglobulin G (IgG), and sheep anti-mouse IgG were from Amersham (Buckinghamshire, UK). A 0.1% dimethyl sulfoxide (DMSO) solution was used to prepare the auraptene suspension.

### 4.2. Platelet Aggregation

This study conformed to the directives of the Helsinki Declaration and was approved by the Institutional Review Board of Taipei Medical University (No. TMU-N201812024). An informed consent form was provided to all human blood donors involved this study. Human platelet suspensions were prepared as previously described [24]. In brief, blood was collected from healthy human volunteers who had taken no medicine during the preceding 2 weeks, and was mixed with acid-citrate-dextrose solution (9:1, *v*/*v*). After centrifugation, the supernatant (platelet-rich plasma; PRP) was supplemented with PGE_1_ (0.5 μM) and heparin (6.4 IU/mL), and then incubated for 10 min at 37 °C and centrifuged at 500× *g* for 10 min. The platelet pellets were suspended in 5 mL of Tyrode’s solution, pH 7.3 [containing (mM) NaCl 11.9, KCl 2.7, MgCl_2_ 2.1, NaH_2_PO_4_ 0.4, NaHCO_3_ 11.9, and glucose 11.1], then apyrase (1.0 U/mL), PGE1 (0.5 μM), and heparin (6.4 IU/mL) were added, and the mixture was incubated for 10 min at 37 °C. After centrifugation of the suspensions at 500× *g* for 10 min, the washing procedure was repeated. Washed platelets were finally suspended in Tyrode’s solution containing BSA (3.5 mg/mL). The final concentration of Ca^2+^ in Tyrode’s solution was 1 mM.

A turbidimetric method was applied to measure platelet aggregation [24], using a Lumi-Aggregometer (Payton, Scarborough, Ontario, Canada). Platelet suspensions (3.6 × 10^8^ cells/mL) were preincubated with various concentrations of auraptene, MG-132, BAY11-7082 or an isovolumetric solvent control (0.1% DMSO, final concentration) for 3 min before the addition of collagen (1 μg/mL). The reaction was allowed to proceed for 6 min, and the extent of aggregation was expressed as a percentage of the control (Tyrode’s solution-treated group) in light-transmission units. While measuring the ATP release, 20 μL of a luciferin/luciferase mixture was added 1 min before the addition of collagen, and assayed using a Hitachi Spectrometer F-7000 (Tokyo, Japan).

### 4.3. Confocal Laser Fluorescence Microscopy

Immunostaining of platelets was carried out as described previously [25]. In brief, resting or collagen (1 μg/mL)-activated platelets (1.2 × 10^9^ cells/mL) were fixed in 4% (*v*/*v*) paraformaldehyde on poly-L-lysine-coated coverslips for 30 min. Platelets were then permeabilized in 0.5% Triton X-100, and incubated with 1% BSA in PBS for 30 min before staining. To observed the p65, platelets were stained with control IgG or anti-p65 mAb for 24 h. After being washed with PBST (PBS + 0.5 Triton X-100), platelets were further incubated with goat anti-rabbit IgG-conjugated fluorescein isothiocyanate (FITC) for 1 h, and p65 was observed under a confocal microscope (Leica TCS SP5, Mannheim, Germany) using a 100 × oil immersion objective.

### 4.4. Immunoblotting Study

Washed platelets (1.2 × 10^9^ cells/mL) were preincubated with auraptene (60 μM) or various reagents for 3 min, followed by the addition of collagen (1 μg/mL) to trigger platelet activation. The reaction was stopped, and platelets were immediately re-suspended in 200 μL of lysis buffer (aprotinin 10 μg/mL, PMSF 1 mM, leupeptin 2 μg/mL, NaF 10 mM, sodium orthovanadate 1 mM, and sodium pyrophosphate 5 mM) for 1 h. Lysates were centrifuged at 5000× *g* for 5 min. Samples containing 80 μg of protein were separated by 12% sodium dodecylsulfate polyacrylamide gel electrophoresis (SDS-PAGE); proteins were electrotransferred by semidry transfer (Bio-Rad, Hercules, CA, USA). Blots were blocked with TBST (10 mM Tris-base, 100 mM NaCl, and 0.01% Tween 20) containing 5% BSA for 1 h and then probed with various primary antibodies. Membranes were incubated with HRP-linked anti-mouse IgG or anti-rabbit IgG (diluted 1:3000 in TBST) for 1 h. Immunoreactive bands were detected by an enhanced chemiluminescence (ECL) system. The bar graph depicts the ratios of semiquantitative results obtained by scanning reactive bands and quantifying the optical density using videodensitometry (Bio-Profil; Biolight Windows Application V2000.01; Vilber Lourmat, France).

### 4.5. Measurement of Hydroxyl Radicals (HO•) by Electron Spin Resonance (ESR) Spectrometry

The ESR method used a Bruker EMX ESR spectrometer as described previously [22]. In brief, platelet suspensions (3.6 × 10^8^ cells/mL) were preincubated with auraptene (30 μM) or BAY11-7082 (10 μM) for 3 min before the addition of collagen (1 μg/mL). The reaction was allowed to proceed for 5 min, followed by the addition of DMPO (100 μM) for the ESR study. The ESR spectrum analysis was performed by using WIN-EPR, version 921201 supplied by BRUKER-FRANZEN Analytik GmbH (Bremen, Germany) [23].

### 4.6. Vascular Thrombus in Mouse Mesenteric Microvessels Irradiated by Sodium Fluorescein

The method applied to the thrombogenic animal model in this experiment conformed to the Guide for the Care and Use of Laboratory Animals (8th edition, 2011), and we received an affidavit of approval for the animal use protocol from Taipei Medical University (No. LAC-2018-0360). In brief, external jugular veins of mice (6 weeks old) were cannulated with a polyethylene (PE)-10 tube for administration of the sodium fluorescein (15 µg/kg) and auraptene (7.5 and 15 mg/kg) intravenously as described previously [26]. Venules (30–40 µm) were irradiated with wavelengths of <520 nm to produce a microthrombus, and the time required for the thrombus to occlude the microvessel (occlusion time) was recorded.

### 4.7. Data Analysis

Experimental results are expressed as the means ± standard error of the mean (SEM) and are accompanied by the number of observations (*n*). Values of n refer to the number of experiments, each made with different blood donors. The significant value among the experimental groups in mice was analyzed by using unpaired Student’s *t*-test. Variations between the experimental setup were calculated using one-way analysis of variance (ANOVA). If this analysis indicated significant differences among group means, then each group was compared using the Student–Newman–Keuls method. *p* < 0.05 was considered statistically significant. Statistical analyses were performed using SAS Version 9.2 (SAS Inc., Cary, NC, USA).

## 5. Conclusions

Herein, we propose a novel inhibitory pathway of NF-κB-mediated PLCγ2-PKC activation by auraptene in human platelets, and further supported that auraptene possesses potent activity for clinical therapeutic or prophylactic application for treatment with thromboembolic diseases. Because platelet activation is linked to hemostasis, and also has a relevant role in inflammation, our present data demonstrating that inhibition of NF-κB interferes with platelet function may have a great impact when these types of drugs are also considered for treating various inflammatory diseases.

## Abbreviations

ATP	Adenosine triphosphate
BSA	Bovine serum albumin
CVDs	Cardiovascular diseases
DAG	Diacylglycerol
DMSO	Dimethyl sulfoxide
ESR	Electron spin resonance
NF-κB	Nuclear factor-κB
PDBu	Phorbol-12,13-dibutyrate
PGE_1_	Prostaglandin E1
PLCγ2	Phospholipase Cγ2
PKC	Protein kinase C
